# Spatial relationship between bone formation and mechanical stimulus within cortical bone: Combining 3D fluorochrome mapping and poroelastic finite element modelling

**DOI:** 10.1016/j.bonr.2018.02.003

**Published:** 2018-02-16

**Authors:** A. Carriero, A.F. Pereira, A.J. Wilson, S. Castagno, B. Javaheri, A.A. Pitsillides, M. Marenzana, S.J. Shefelbine

**Affiliations:** a Department of Biomedical Engineering, The City College of New York, New York, NY, USA; b Department of Bioengineering, Imperial College London, UK; c Graduate School of Biomedical Engineering, University of New South Wales, Australia; d Department of Life Science, Imperial College London, UK; e Department of Medicine, Imperial College London, UK; f Department of Comparative Biomedical Sciences, Royal Veterinary College, UK; g Department of Mechanical and Industrial Engineering and Department of Bioengineering, Northeastern University, Boston, MA, USA

**Keywords:** Bone adaptation, 3D fluorochrome mapping, Cortical bone, Mouse, Tibia

## Abstract

Bone is a dynamic tissue and adapts its architecture in response to biological and mechanical factors. Here we investigate how cortical bone formation is spatially controlled by the local mechanical environment in the murine tibia axial loading model (C57BL/6). We obtained 3D locations of new bone formation by performing ‘slice and view’ 3D fluorochrome mapping of the entire bone and compared these sites with the regions of high fluid velocity or strain energy density estimated using a finite element model, validated with ex-vivo bone surface strain map acquired ex-vivo using digital image correlation. For the comparison, 2D maps of the average bone formation and peak mechanical stimulus on the tibial endosteal and periosteal surface across the entire cortical surface were created. Results showed that bone formed on the periosteal and endosteal surface in regions of high fluid flow. Peak strain energy density predicted only the formation of bone periosteally. Understanding how the mechanical stimuli spatially relates with regions of cortical bone formation in response to loading will eventually guide loading regime therapies to maintain or restore bone mass in specific sites in skeletal pathologies.

## Highlights

• 3D spatial representation of new bone formation after loading is shown by fluorochrome mapping of the entire mouse tibia
• Regions of new bone formation spatially associate with regions of high strain and fluid mechanical stimulus in a FE model
• The FE model was validated with the strains on the bone surface determined ex-vivo using digital image correlation
• Regions of new bone formation co-localize in sites of peak fluid flow, both endosteally and periosteally
• Peak strain energy density was able to predict only periosteal bone formation

## 1 Introduction

Though it has been recognized for centuries that bone adapts its architecture in response to applied loads (Wolff, 1892), we still do not understand how the process is spatially regulated. In particular, it is not well known how the local mechanical environment correlates with regions of bone apposition. Does bone adaptation occur where a particular mechanical stimulus is high? What is that mechanical stimulus?

We currently have limited understanding of the spatial regulation of mechanoadaptation because: 1) it is challenging to accurately investigate a spatially and temporally varying mechanical field in the bone during loading; and 2) it is difficult to identify regions of adaptation throughout the bone, when the amount of local bone formation can be on the order of 10 μm or less. Previous studies have used a combination of finite element modelling, in vivo microCT imaging, and bone histomorphometry to correlate the mechanical environment and regions of cortical bone formation (Webster et al., 2012; Razi et al., 2015a; Moustafa et al., 2012; Birkhold et al., 2017; Gross et al., 1997; Judex et al., 1997). In a mouse vertebral loading model, it was found that strain energy density averaged within a cross-sectional regions of cortical bone predicted regions of bone formation along the length of the vertebrate (Webster et al., 2012). However, these studies did not identify how bone adaptation and mechanical stimuli within a cross-section related to each other in specific locations. In a turkey radius loading model (Gross et al., 1997), peak circumferential strain gradients (which closely relates to fluid flow), calculated with a finite element modelling in 24 sectors of a single mid-diaphyseal section, strongly correlated with the specific regions of periosteal bone formation while strain energy density did not. Similarly, peak strain gradients correlated with sites of periosteal bone formation in an exercise-induced rooster model (Judex et al., 1997). More recently, in the mouse tibial loading model, “4D” in-vivo microCT was used to identify regions of bone formation and resorption from sequential in-vivo scans in a region of interest spanning 5% of the tibial length (Birkhold et al., 2015; Birkhold et al., 2016). For each modelling event, the principal strains in that region (~10 μm) were determined from a finite element model (Birkhold et al., 2016). Regions of bone formation correlated with high principle strain at the periosteal surface, but not at the endosteal surface (Razi et al., 2015a; Birkhold et al., 2016). Other studies using the mouse tibial loading model concluded that there is no correlation between regions of high peak longitudinal strain and bone formation in tibial cortical bone when the stimuli was compared to regions of bone formation in two specific histomorphometric sections (37% and 75% of the bone length) (Moustafa et al., 2012).

These previous studies indicate a possible spatial relationship between local mechanical stimuli and cortical bone adaptation. However, there are limitations in relying on in-vivo microCT with nominal isotropic resolution of 10 μm to detect events occurring on the length scale of 10 μm. Also, examining only a small portion of the bone or a few cross-sectional areas, and only the periosteal surface limits the ability to draw conclusions about the relationship between the mechanical environment and bone formation throughout the entire cortical bone. In addition, previous studies only examined strain-dependent stimuli (strain energy density, longitudinal strain, and principal strain), which capture bone's sensitivity to load magnitude but not load rate, and do not predict endocortical formation events, as the stimulus is maximum on the outer surface. Time dependent stimuli (such as fluid velocity) capture the rate-dependent effects, periosteal and endocortical bone formation (Pereira et al., 2015) and are related to shear stresses at the osteocytic level (Weinbaum et al., 1994; You et al., 2001). Thus, in this study we use the murine tibial loading model to determine spatial relationships between the mechanical environment and bone formation in the tibial cortical bone. We examine both strain and fluid flow dependent stimuli in a finite element model. Regions of bone formation are detected with three-dimensional imaging of fluorochrome labels in a novel slice-and-view technique for cortical bone histomorphometry. The mechanical stimuli (strain energy density or fluid velocity) are compared visually and quantitatively to regions of bone formation.

## 2 Materials and methods

### 2.1 In-vivo tibial loading model

The right leg of five female C57BL/6 mice (22 week old, Charles River Company, UK) was loaded with a custom tibial loading rig (De Souza et al., 2005). The left leg was not loaded and used as control. In this study, trapezoidal load cycles were applied for 0.1 s, with a peak load of 12 N and a rest period of 10 s, using a regime of 40 cycles a day, 3 times a week for 2 weeks (De Souza et al., 2005). Calcein was administered intraperitoneally once (on day 5), which was about one-third of the way through the experimental period (14 days). Mice were sacrificed on day 15. Mice were maintained under standard laboratory conditions and experiments were conducted in compliance with the ARRIVE (Animal Research: Reporting of In Vivo Experiments) guidelines for reporting. Briefly, mice were housed up to 4 per cage in polypropylene cages with wood chip and paper bedding and provided standard rodent maintenance diet (Special Diet Services, South Witham, UK) and water ad libitum throughout the study. All procedures complied with the UK Animals (Scientific Procedures) Act 1986 and were reviewed and approved by UK Home Office and local ethics committee of the Royal Veterinary College (London, UK).

### 2.2 Strain map on the bone surface using digital image correlation

Right and left tibiae of the five mice subjected to the mechanical loading protocol were considered. Tibiae were exposed by removing soft tissues, and a thin layer of matt, water-based, white paint with subsequent matt, acrylic, black ink speckles was applied using a high precision air brush (Carriero et al., 2014). Speckles were randomly distributed with a 45% black/white density and dots of about 8 pixels in diameter (Carriero et al., 2014). Legs were inserted in the loading cups and loaded to 12 N to replicate the load in vivo (Instron 5800, High Wycombe, UK) while two CCD cameras (100 mm lenses, GOM GmbH, Germany) mounted on a tripod recorded images of the medial side of the tibiae surface with a resolution of 7.5 × 10.9 μm at 1 N interval (GOM GmbH, Germany) (Carriero et al., 2014). Square facets (19 × 19 pixels) with 15 pixels step facet were used for the post-processing of the images using ARAMIS 5 M System (GOM GmbH, Germany) (Carriero et al., 2014). Paired images were taken in the undeformed state to determine the amount of experimental error (noise), and the surface of each tibia were imaged at least two times to demonstrate repeatable strain fields. These ex-vivo strain maps determined strain magnitudes and distribution patterns in mature tibiae with and without prior adaptation to applied load (right and left tibia, respectively). Peak and average strain at 12 N on the medial surface of the control non-adapted (left) leg and the load adapted (right) leg were calculated, averaged across the specimens of each group, and statistically compared. Homogeneity of variance and normality of the variables were assessed using the Levene's test and the Shapiro-Wilk test, respectively (SPSS, IBM, Somers, NY, USA). Difference between the two groups was assessed using paired *t*-test. Statistical significance was set at *p* < 0.05.

### 2.3 Bone architecture using 3D Micro-Computed Tomography

After determination of surface strains across the entire tibia by DIC, bones were dissected and scanned by microCT at 10 μm resolution. Images were acquired using a Skysan 1172 micro-CT system (Skyscan, Kontich, Belgium) with the x-ray tube operated at 50 kV, 200 μA, 1600 ms exposure time with a 0.5 mm aluminium filter and a focal spot size of 5 μm. The microCT images were imported into image-processing software (Mimics V15, Materialise, Leuven, Belgium). Tibiae were segmented, (with fibula removed from the image) and aligned along their longitudinal axis. Minimum moment of area (Imin,) along the tibial diaphysis was determined using ImageJ (Schneider et al., 2012; Pereira, 2014). We previously determined Imin was more sensitive to regions of adaptation than Imax because of the location of regions of adaptation and in relation to the principle axes (Pereira, 2014). The longitudinal distance between the proximal and distal tibia-fibula junctions were normalised for all bones.

Cross-sectional morphology from microCT of the loaded and unloaded tibiae identified general regions along the length of the bone where adaptation occurred. Box plots were used to represent the minimum moment of area along the entire length of the tibia. Statistical significant difference between load adapted and control leg was considered when no overlap exists between the confidence interval (CI) of the blocks of the two groups.

### 2.4 Location of bone formation using 3D fluorochrome mapping

**Fig. 1:**
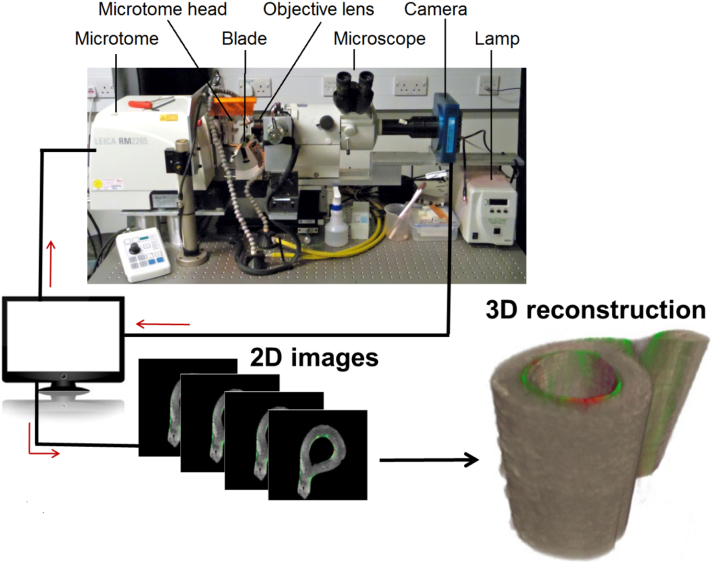
Schematic of the automatic imaging acquisition and bone reconstruction used during 3D fluorochrome mapping. The histocutter is a combination of the microtome for automatic cutting and a fluorescence microscope for image acquisition. The fluorescence microscope has UV, green, and red emission filters. A grey scale CCD camera positioned behind the fluorescence microscope captures the fluorescence light emitted by the sample. The distance between the camera and the exposed surface is fixed to ensure a constant focus through a series of slicing and imaging process. An in-house custom written code synchronizes the microtome action, filter wheels, shutters, and the camera operation for capturing the imaging that is then sent to a hard disk for storage. In this study, we used a 3.3-x objective with a 1.591 μm in-plane resolution and a field of view of 4.585 mm. We imaged the bone sample using an ultraviolet (UV) excitation filter (excitation 365, emission 450; Nikon), the calcein fluorescence using a green filter (excitation 480, emission 535; Nikon) and alizarin red using a red filter (excitation 560, emission 645; Nikon). About 3000 sections were imaged at a distance of 5 um for each bone, thus collecting 6000 images. (For interpretation of the references to colour in this figure legend, the reader is referred to the web version of this article.)

Loaded and control tibiae of one mouse were then fixed and embedded in an opaque methyl-methacrylate (PMMA) containing Sudan Black dye (2%) to preserve calcein labels and provide an opaque embedding material to block out fluorescence behind the plane of imaging. Each embedded bone block was then mounted in the “histocutter” (Fig. 1) that allows serial cutting (Leica RM-2265 Microtome) and imaging (Nikon AZ-100 Fluorescence Microscope) of the ‘block face’ in order to create a 3D histological reconstruction (3.3× projection lens, zoom 2, 4.585 mm field of view), similar to what 3D techniques used for trabecular bone in other groups (Kazakia et al., 2007; Bigley et al., 2008; Goff et al., 2012; Goff et al., 2014; Matheny et al., 2013; Slyfield et al., 2009; Slyfield et al., 2012a; Slyfield et al., 2012b; Tkachenko et al., 2009). The microscope was equipped with a wheel with multiple emission filters (Nikon, UK) to allow imaging at different wavelengths so that different fluorescence labels and ultraviolet light can be captured from the cut face after each serial section. We used green (excitation 460–500 nm and emission 510–560 nm) and ultraviolet (excitation 348–383 nm and emission 420–480 nm) filter cubes, to allow for the visualization of calcein label and mineralized bone, respectively. A CCD camera (Apogee Alta U16 M) imaged each serial face in a ‘slice and view’ routine, generating a stack of 2D images. The process was optimized so that images had reasonable resolution and processing time was convenient for the entire mouse tibia to be scanned. Images were collected with a nominal resolution of 1.591 μm in the lateral direction and 5 μm in the axial direction, corresponding to the slice cut thickness. Approximately 3000 images were acquired per filter per tibia, requiring 20 h of imaging acquisition for one bone. They were then processed using ImageJ (Schneider et al., 2012) and reconstructed to make a 3D map of bone formation with Amira (FEI Corporation, USA). These were then compared to the strain map on the bone and its morphology from the microCT analysis for both control non-adapted and load adapted legs.

### 2.5 3D strain maps and location of bone formation using finite element modelling

Fluid-flow in the lacunar-canalicular porosity is thought to be the prime actuator triggering anabolic mechanotransductive pathways on bone cells (Weinbaum et al., 1994). Poroelastic theory can provide predictions of lacunar-canalicular fluid flow velocity at a continuum level. In previous predictive studies of bone adaptation of the mouse tibia to axial loading, we showed that load-induced fluid velocity is an accurate mechanical indicator of local remodelling activity in an 8 week old mouse (Pereira et al., 2015). We used similar poroelastic finite-element models in this study to estimate strains and fluid flow velocities experienced by a 22 week old mouse tibial bone during axial loading.

A four-node tetrahedral surface mesh was imported from Mimics into Abaqus/CAE (v6.12, ABAQUS Inc., Pawtucket, RI, USA). A volume mesh was computed and a convergence study on the mesh density was performed (Pereira, 2014). Elements with a pore pressure degree of freedom were considered to account for fluid flow inside the lacunar-canalicular space. Periosteal and endosteal membranes were modeled as element layers with isotropic and elastic material properties similar to fibrous tissue (Lacroix and Prendergast, 2002). A periosteal Darcy permeability of 10^−17^ m^2^, as measured by Evans et al. (2013), was used in both membrane layers, i.e. periosteal and endosteal surfaces were assumed to have the same permeability (Pereira et al., 2015). Material properties of the bone and cartilage compartments were assigned as described by Pereira et al. (2015) (Table S1, Table S2).

Loading conditions were determined from a study by Poulet et al. (2011) where microCT imaging was employed to visualize the knee joint during axial loading. Axial load was simulated as concentrated forces distributed on a set of nodes at the proximal condyles. The loads resulted in a total of 12 N applied as a single trapezoidal load cycle. A set of nodes at the distal interior articular surface was fixed. Half a second of total time period was calculated, with solver time step set to 0.005 s. Two stimuli were examined: strain energy density and fluid velocity. Fluid velocity magnitude was calculated using standard soils consolidation formulations and Darcy's law in which fluid velocity is proportional to the gradient of pore pressure. For each stimulus we compared the amount of stimulus with the bone formation regions from the 3D fluorochrome mapping.

### 2.6 Quantification of simulations accuracy in predicting 3D bone formation

To compare regions of bone formation with the mechanical stimulus, 2D spatial maps quantifying the amount of bone formation and mechanical stimuli were generated. The 3D tibiae from the histocutter and the finite element model were registered together. Between the proximal and distal fibular junctions, each tibia was “unwrapped” into a polar representation with a line to the centroid of the fibula at 0°. By considering 45 8° pies along the long axis of the bone, calcein label intensity and peak fluid velocity representing location of bone formation during mechano-adaptation on the periosteal and endosteal surfaces were calculated on each bone cross-section by averaging the value on each surface within each angular wedge. Each of these values constituted (i) a point in the plot of the average fluorochrome intensity or stimulus vs. polar coordinate frame for each slice, and (ii) a pixel value in the 2D spatial map of the bone formation on the endosteal and periosteal surface from either the fluorochrome images or the FE estimated mechanical stimulus. The qualitative comparison of these maps allowed the investigation of spatial association between regions of bone formation and mechanical stimulus.

## 3 Results

### 3.1 Digital image correlation

**Fig. 2:**
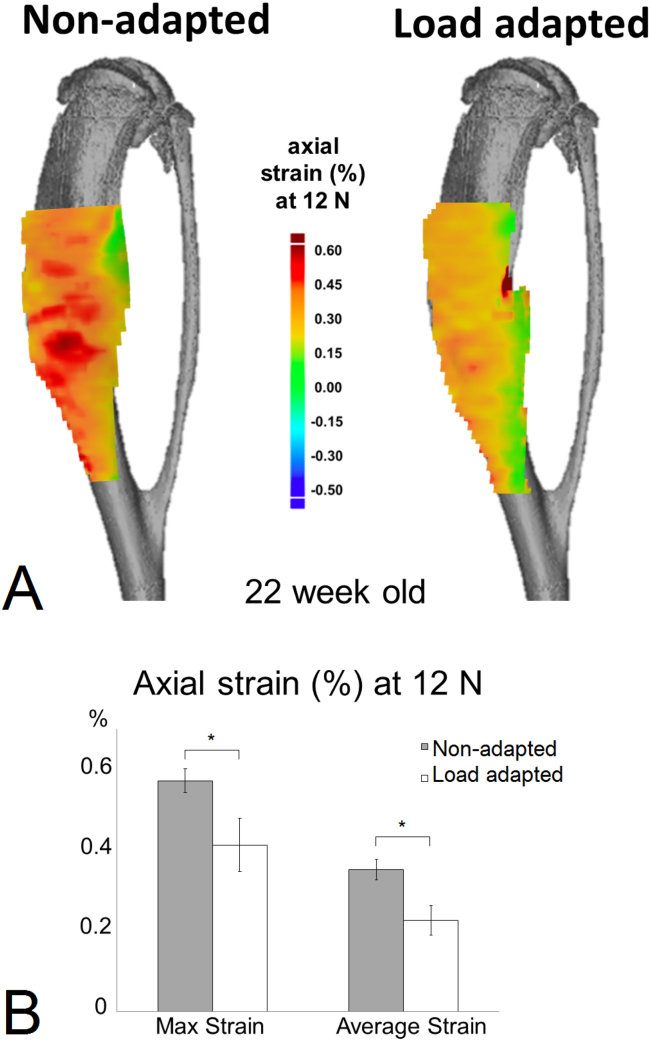
(A) The strain map on the bone surface at 12 N of load assessed with a DIC system on a representative control and loaded 22 week-old mouse tibia (images for the load adapted tibia are reflected to allow a direct comparison with those of the control, non-loaded tibia). (B) Peak and average strain measured on the bone surface of 5 control and 5 loaded samples at 12 N. (^⁎^ indicates *p* < 0.001).

The spatial strain distribution over the medial side of the tibia was measured, and only the strain variation in the axial (loading) direction is reported as transverse and shear strains had relatively low magnitude in comparison (Fig. 2A). Similar to our previous studies in 8 (Sztefek et al., 2010), and 12 and 18 week (Carriero et al., 2014) old males, the 22 week old female control non-adapted tibiae exhibited non-uniform load-induced surface strain fields (Fig. 2A), with tension on the medial side and compression on the lateral side. Local regions of high strain (>0.5%) were seen on the medial side of the control non-adapted tibiae. Load-related tissue strain significantly decreased in the adapted leg after two weeks of loading (Fig. 2B). The medial side of the control non-adapted tibia had a peak and a mean strain of 0.571% ± 0.35 and 0.351% ± 0.03, respectively. These decreased following in-vivo adaptation after 2 weeks of applied loading to 0.411% ± 0.23 and 0.225% ± 0.04.

The noise was consistent throughout all the tests and was approximately 0.03%. No failure during the load was observed, as the load-deformation curve was similar during each repeated loading episode.

### 3.2 Micro-Computed Tomography

**Fig. 3:**
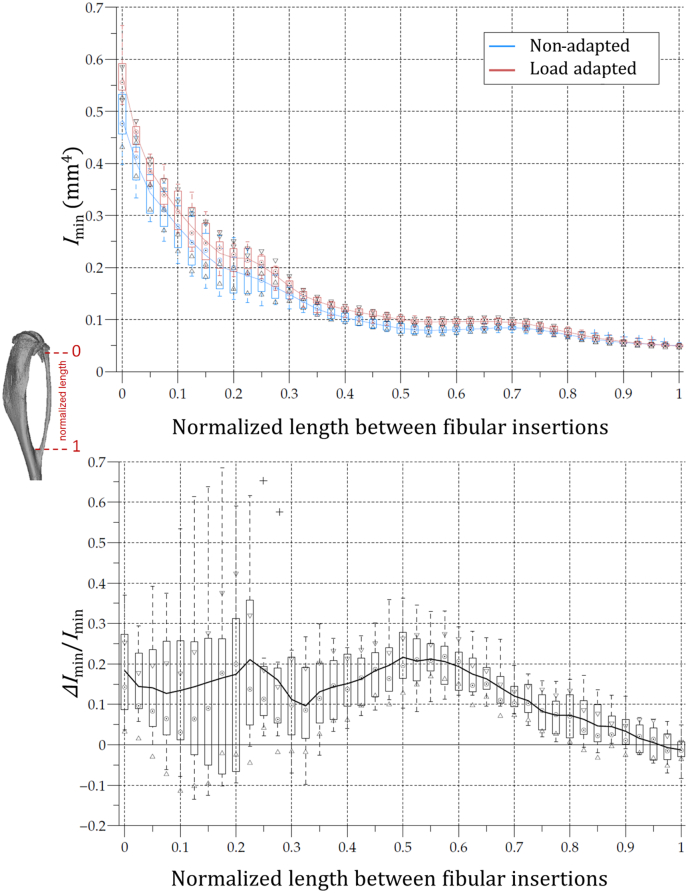
Boxplot representation of the 22 w.o. mouse tibial second moment of area about the minor axis (I_min_) of the control (blue) and loaded (red) legs as a function of the normalised diaphysial length between the fibular insertions. I_min_ of the non-adapted leg was used to normalize the ΔI_min_. (For interpretation of the references to colour in this figure legend, the reader is referred to the web version of this article.)

MicroCT scans of the right (load adapted) and left (non-adapted) legs were compared for cross-sectional properties along the length of the bone. I_min_ was significantly larger in the load adapted tibia compared to the control non-adapted bone, particularly over the proximal 20–40% tibial length (Fig. 3). Very few differences were found in the distal portion of the tibia.

### 3.3 3D fluorochrome mapping

**Fig. 4:**
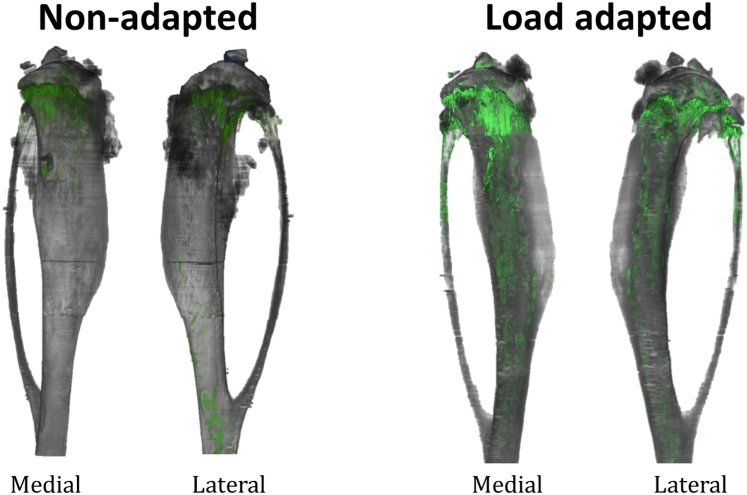
3D reconstruction of a representative 22 w.o. mouse tibia bone (in grey) from a control (unloaded leg) and a loaded leg showing the calcein label on the medial and lateral surface of the bone (in green). The calcein label here depicts where bone has been formed only on the external surface of the bone, in two week's time frame in the control leg and in the leg loaded with our regime. These models are made of about 3000 histological slices (raw images) per fluorescence filter stacked together to make a 3D composite image of the bone and of its calcein label showing exactly where bone formation is happening without relying on any registration algorithm. Images for the loaded leg are reflected to allow a direct comparison with those from the control leg. (For interpretation of the references to colour in this figure legend, the reader is referred to the web version of this article.)

**Fig. 5:**
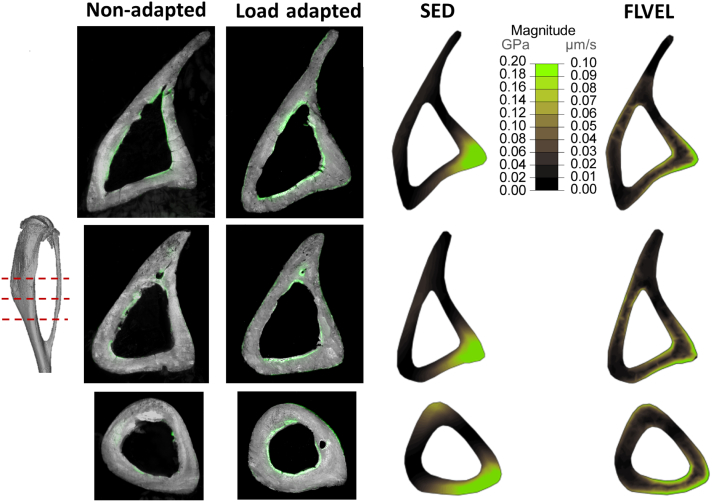
Bone histomorphometry slices showing regions of bone adaptation and FEM slices showing regions of high stimulus taken at 3 locations at the mid-shaft of the bone (tibial bone is in grey and bone growth is represented in green). Bone fluorescence mapping of the same mouse tibiae represented in 3D in Fig. 4 and Fig. 7 shows the bone formation on the endosteal and periosteal surfaces of the bone (image resolution is 1.591 μm × 1.591 μm × 5 μm - images for the loaded leg are reflected to allow a direct comparison with those from the control leg). Regions of high stimulus due to loading: strain energy density (SED) only has high stimulus on the periosteal surface, while regions of high fluid flow (FLVEL) are both periosteally and endosteally. M and L indicate the medial and lateral side of the tibia, respectively. (For interpretation of the references to colour in this figure legend, the reader is referred to the web version of this article.)

We obtained high resolution imaging of bone adaptation for our 22 week-old mouse bones. Video S1 in the supplementary material shows the regions of bone adaptation highlighted with green and the bone image from the ultraviolet signal in grey along the entire bone length for the load adapted and the non-adapted tibial bone. The stacks of 2D images were then reconstructed in 3D composite images (Fig. 4) to reveal regions of bone formation (calcein labels) overlaid on the unprocessed ultraviolet signal. In the non-adapted tibia there is very little, mostly endosteal, cortical bone formation. In the loaded tibia, bone adaptation occurred on the medial and lateral sides of the endocortical surface (Fig. 5) and on the proximal half of the periosteal surface (Fig. 4). Trabecular remodelling is seen in the loaded and unloaded legs (Fig. 4).

### 3.4 Finite element modelling

**Fig. 6:**
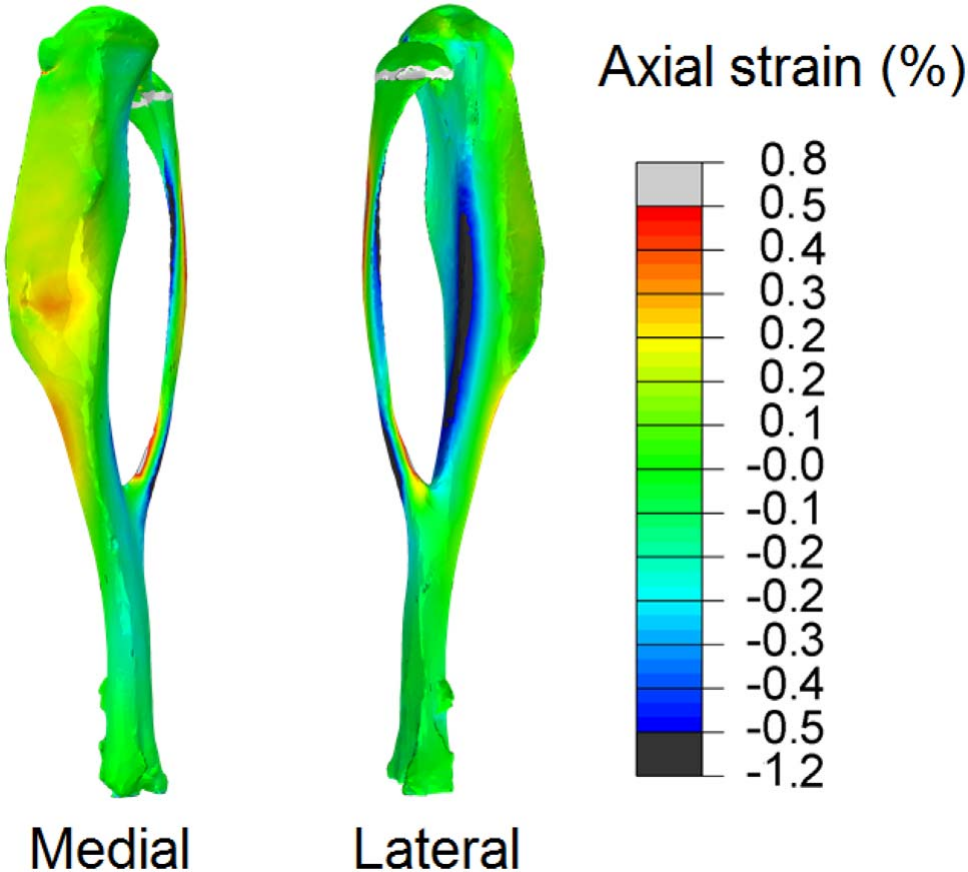
The strain map on the bone surface at 12 N of load assessed using a finite element model of a representative control 22 w.o. mouse tibia bone, with homogeneous isotropic material properties, have similar distribution as the strain obtained experimentally using DIC (Fig. 2A).

Fig. 6 reports the strain map on the entire tibia of a 22 week-old mouse obtained from the finite element analysis. The distribution of longitudinal strains is in close agreement with those obtained ex-vivo with the DIC, with a high tensile strain peak forming on the medial surface of the bone. In contrast, the lateral side of the tibia shows high compressive strain, similar to those already encountered in our previous experimental studies on tibiae from 10 week-old mice (Sztefek et al., 2010). However, the magnitude of the strain is somewhat lower than those measured by our experimental DIC results, most likely because of the homogeneity of materials and loading assumptions of our model. In this study the relative patterns of strain are of primary interest, the absolute magnitude is less critical.

**Fig. 7:**
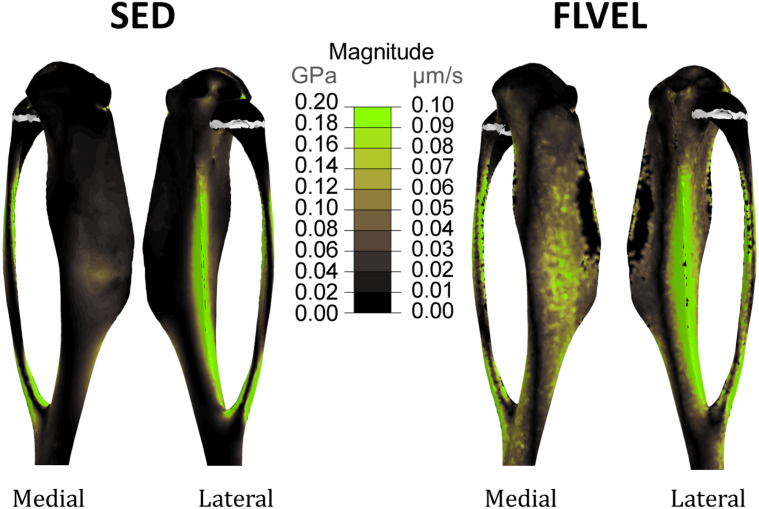
3D representation of the location of new bone formation (in green) on a representative 22 w.o. mouse tibia bone (in grey) from a control (unloaded leg) due to strain energy density (SED) fluid flow (FLVEL) stimulus elicited by the strain distribution at 12 N represented in Fig. 2. The calcein label here depicts where bone has been formed only on the external, medial and lateral, surface of the bone. (For interpretation of the references to colour in this figure legend, the reader is referred to the web version of this article.)

Fig. 7 shows the magnitude of strain energy density and fluid velocity across the tibial bone geometry at the time of peak loading (*t* = 0.025 s). Regions of high strain energy density are only located periosteally, failing to predict the endosteal bone formation induced by loading of the tibia (Fig. 5). Regions of high fluid velocity are, instead, present in the medial central part of the tibia and along the lateral crest of the bone, also where we demonstrate bone formation to be located in the 3D fluorochrome mapping (Fig. 5).

### 3.5 Simulation accuracy

**Fig. 8:**
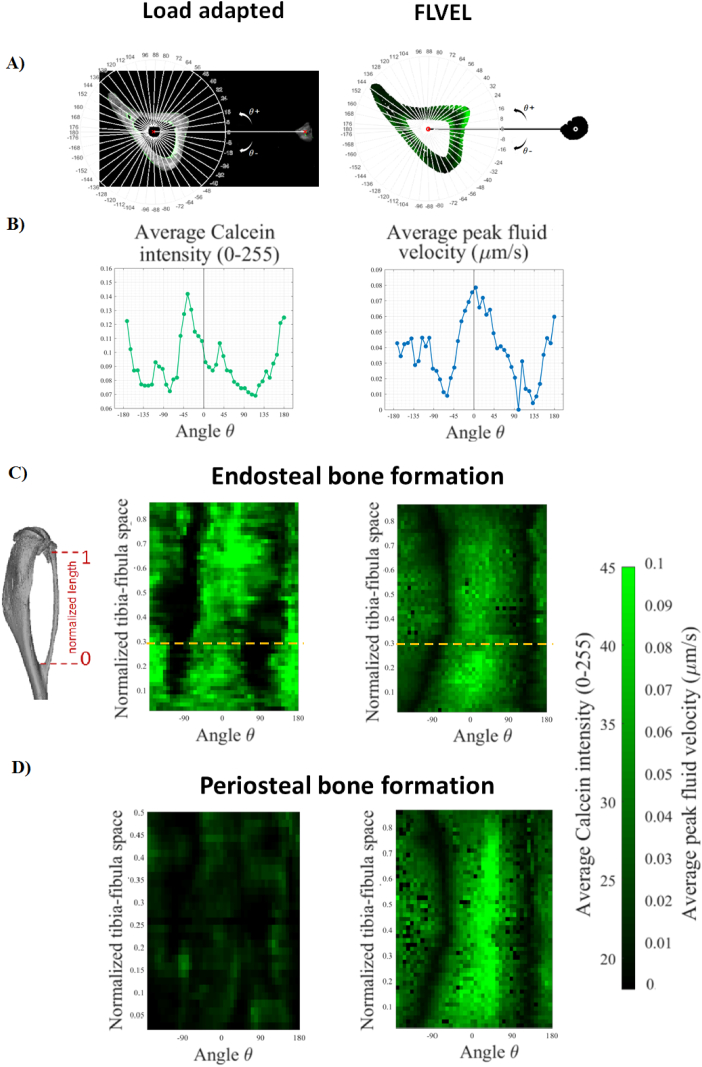
(A) Single cross-section of the tibia with the fibula for the bones used for the load adapted fluorochrome mapping and the bone used for the finite element modelling. The image shows the construction of the polar coordinate system where the 0° corresponds to the axis connecting the center of the fibula and the center of the tibia. (B) Average calcein density and average peak fluid flow velocity on the endosteal surface calculated in each 8° pie along the radial direction for the cross-sections of bones reported in (A). Each point value corresponds to the average value calculate in a radius of 0.1 mm around the surface line. (C–D) 2D “unwrapped” bone formation maps along the tibia-fibula junctions for the 3D fluorochrome and the fluid flow FE model on the endosteal and periosteal surface, respectively. The orange lines on the endosteal bone formation maps indicate the values calculated for the cross-section in (A).

Fig. 8 reports colour maps of average calcein intensity from the 3D fluorochrome mapping and the average peak fluid velocity obtained from the computational modelling of mechano-adaptation on the endosteal and periosteal surfaces. Our mapping shows agreement in the location of bone adaption on both endosteal and periosteal surfaces, with the majority of the bone formation (stimulus) on the lateral side (around 0°), very little bone formation (stimulus) anterior or posterior (±90°), and moderate bone formation (stimulus) medially (180°).

## 4 Discussion

This work uses a combination of novel high-resolution techniques to investigate the spatial distribution of the bone mechano-adaptative response on the entire cortex by directly visualizing the locations of bone formation in a murine tibia loading model using 3D fluorochrome mapping. Here, we further determine the relation between regions of bone adaptation and locations of high mechanical stimuli (i.e. strain energy density and fluid flow) assessed using FE computational models experimentally validated with DIC bone surface strain mapping ex-vivo. Our results show that in adult mouse (i) bone adapts to loads along its entire length on both the endosteal and periosteal surface, and (ii) high fluid flow is able to predict formation of bone on both these surfaces, while high strain energy density can predict bone formation only periosteally. Providing clues to the biophysical stimuli to which bone responds, future studies can use this knowledge to develop loading protocols that direct bone adaptation to a specific site.

Previous studies using the tibial loading model and 4D microCT in adult mice found bone adaptation on both periosteum and endosteum, with greater bone formation on the endosteal surface (Birkhold et al., 2017; Birkhold et al., 2016), in agreement with our study. However, these studies only investigated the association of bone adaptation to strain driven mechanical stimuli and concluded that bone adapts at the periosteal surface in locations of high principal strains while it adapts at the endosteal surface in locations of low principal strains. In this study we investigated both strain and fluid flow driven mechanical stimuli and found a good spatial agreement between regions of new bone formation (i.e. average calcein intensity) and peak of fluid velocity predicted with FE models on both endosteal and periosteal surfaces. Previous studies found similar results when examining the correlation between mechanical stimuli and bone formation on the periosteal surface in a single cross-section of the bone (Gross et al., 1997; Judex et al., 1997). Here, we are able to predict the spatial association of fluid flow with bone formation on its entire length and on both periosteal and endosteal surface. In this study peak strain energy density was only able to predict periosteal bone formation. When comparing experimental data vs. computational prediction of bone formation, we do not expect exact one-to-one mapping as the bone in the FE model is necessarily different (a control leg) than the bone used for histological imaging of adaptation (a loaded leg). Therefore, it would not be appropriate to quantify the exact spatial correlation of two different bones. Instead we focus on regions of adaptation longitudinally and circumferentially in both the periosteum and endosteum.

In our fluid flow FE modelling we predicted bone formation periosteally, showing a similar trend of our periosteal labels (Fig. 8D), which was present (as evidenced on the composite full bone image in Fig. 4) but interestingly were very weak compared to the endosteal labels. In standard histomorphometry the intensity of the label is not taken into account and only considered as present or absent. In periosteal adaptation figures (Fig. 8C–D) the intensity of the adaptation pattern does not match, although they show similar trend, indicating strong adaptation along the length at 0° (the lateral surface). To make the FEA prediction reflect more accurately the periosteal label, we could decrease periosteal permeability, which would limit periosteal fluid flow and amount of predicted adaptation. Future studies will be needed to further investigate membrane permeability in bone experimentally, and define the role of permeability on the prediction of bone formation due to loading using histomorphometry. However, the general regions of adaptation are spatially predicted by our model.

The combination of the techniques here utilized for the study of bone adaptation is very innovative and powerful. Our study is the first to use DIC strain mapping, FEA prediction of mechanical stimulus, and 3D fluorochrome mapping of bone formation to determine spatial relations between the mechanical environment in the bone and cortical adaptation. These methods are a significant advance over the standard measurements, in which the mechanical stimuli is characterized by a strain gauge measurement and bone formation amounts are measured in a single histological slice at mid-shaft. Previous studies have examined correlations of mechanical stimuli and bone formation within a single cross-section (Gross et al., 1997; Judex et al., 1997) or in only a small section of cortical bone (Razi et al., 2015a) and only a few of these examined endosteal bone formation. These previous studies often used stimuli insensitive to loading rate and frequency (such as strain energy density) (Moustafa et al., 2012; Patel et al., 2014; Razi et al., 2015b; Yang et al., 2014), which limits the ability to determine spatial and temporal influences on adaptation. We demonstrate here that when the finite element model is validated and mechanical parameters other than strain magnitude are examined, the regions of bone formation are predicted by the local fluid velocity especially at the endosteal surface, which is not captured by strain-dependent stimuli.

Standard microCT analyses of cross-sectional parameters, which are typically reported in adaptation studies (De Souza et al., 2005; Holguin et al., 2014; Main et al., 2014; Poole et al., 2012; Robling et al., 2002; Weatherholt et al., 2013; Willinghamm et al., 2010), indicate regions along the tibia cortex where bone formation is high, but do not indicate where within the cross-section these adaptations occur. Recent studies have registered bone in the loaded limb with the contralateral non-loaded limb at each cross-section (Pereira et al., 2015) or with previous in-vivo scans of the same bone (Birkhold et al., 2015; Birkhold et al., 2014; de Bakker et al., 2015; Silva et al., 2012; Lambers et al., 2012; Schulte et al., 2011; Lukas et al., 2013). However, these methods are limited by the accuracy of the registration, radiation exposure, and the resolution of the scan (de Bakker et al., 2015; Lan et al., 2013). 3D fluorochrome mapping allows direct visualization of regions of active bone formation on the entire mouse tibia at a very high resolution. The resolution used in our 3D fluorochrome mapping is much higher (1.591 μm for our images) than the resolution used in in-vivo microCT-based histomorphometry (10 μm), thus we are able to more accurately identify small regions of bone formation. In this study on adult mice we show the results from only one fluorescence label and the UV channel for natural collagen fluorescence; however, we demonstrate the possibility of a multi-label usage in our preliminary data (Fig. 1). The use of multiple labels, such as alizarin red or orange XO, is also possible if injected at different time frames during the treatment period. Future studies will explore the optimisation of these multiple labeling methods for 3D histomorphometry.

3D fluorochrome mapping is not a high through-put technique. Here we demonstrate the technique on a representative pair of bones (loaded and unloaded), resulting in the equivalent of over 3000 histological sections per each bone. Large fields of view (entire bone) at high resolution (1.5 μm) necessarily come at the cost number of samples analyzed. Here we use histological sections of one entire bone to represent adaptation in the tibial loading model. Traditionally studies have used a single histological section to represent the adaptation in the entire bone, but used multiple animals. To obtain spatial mapping of bone adaptation, it is critical to have a full map. Analyzing multiple bones with 3D fluorochrome mapping will be a necessary next step to understand spatial reproducibility of bone adaptation. However, analysis of multiple bones would not likely provide more information about how regions of adaptation correlate with the mechanical environment.

One benefit of classic bone histomorphology is quantification of the amounts of bone formed, the rates of bone formation, and the percentage of active bone surface. In this study we did not quantify the amount of bone formation, which is given by the distance of two consecutive labels. We were limited by only one label, which is fully sufficient to see regions where bone is active. However, the single label had a different thickness and intensity on the periosteal and endosteal surface of the same bone, which may indicate regions of more (endosteally) or less (periosteally) bone formation in the same bone (Fig. 8C–D). In future work quantitative parameters of 3D bone formation will be derived from double-labeling bones using this technique.

### 4.1 Conclusions

In this study we combined in-vivo tibial loading, DIC bone strain maps, FE modelling, and 3D fluorochrome mapping of the murine tibia to provide insight into the mechanical stimuli promoting the spatial regulation of bone formation and osteogenic activity. Our results indicate there is potential to eventually use targeted loading to increase local bone fluid flow and direct bone formation to a defined region. Understanding how to control, direct, and optimize spatial adaptation of bone will help to inform therapy regimes for maintaining bone mass.

The following are the supplementary data related to this article.

Video S1 3D fluorochrome mapping of entire bone showing the location of new bone formation in control (non-adapted) and load-adapted tibia of a 22-week-old mouse. New bone formation is presented with green calcein label in the mouse tibia as we slide through the entire bone. In the non-adapted leg (on the left) very little bone is adapting under normal loading regime. The loaded leg (on the right) instead displays much more green label on the bone surface showing areas of bone formation due to the regime load implemented in this study. Video S1

Table S1 Material properties used for the 3D finite element modelling of the tibia taken from Pereira et al. (2015). Table S1

Table S2 Interstitial fluid material properties used for the 3D finite element modelling of the tibia taken from Pereira et al. (2015). Table S2
