# Supplementary material for: Spatial relationship between bone formation and mechanical stimulus within cortical bone: Combining 3D fluorochrome mapping and poroelastic finite element modelling
Source: Bone Rep. 2018 Feb 16;8:72–80. doi: 10.1016/j.bonr.2018.02.003 (PMC5997173; doi:10.1016/j.bonr.2018.02.003)
Supplement: Table S1 — Material properties used for the 3D finite element modelling of the tibia taken from Pereira et al. (2015). [file mmc2.docx]

**Supplementary Material**

| Property |  | Units | Bone | Membrane layers | Growth plate elements |
| --- | --- | --- | --- | --- | --- |
| Longitudinal Young’s modulus | *E_z_* | MPa | 17 x 10^3^ | 2 | 10 |
| Transverse Young’s modulus | *E_t_* | MPa | 11.5 x 10^3^ | 2 | 10 |
| Transverse Poisson’s ratio | *ν_t_* | - | 0.38 | 0.167 | 0.167 |
| Longitudinal-transverse Poisson’s ratio | *ν_lt_* | - | 0.41 | 0.167 | 0.167 |
| Transverse shear modulus | *G_t_* | MPa | 4.1 x 10^3^ | 0.73 | 6.86 |
| Longitudinal-transverse shear modulus | *G_lt_* | MPa | 5.2 x 10^3^ | 0.73 | 6.86 |
| Pore volume fraction | *Φ* | % | 5 | 0.8 | - |
| Longitudinal permeability | *K_l_* | m^2^ | 1 x 10^-22^ | 1x10^-17^ | - |
| Transverse permeability | *K_t_* | m^2^ | 1 x 10^-23^ | 1x10^-17^ | - |
| Effective stress coefficient | *α* | - | 0.14 |  |  |

**Table S1.** Material properties used for the 3D finite element modelling of the tibia taken from Pereira et al. ^(38)^
