# Supplementary material for: Spatial relationship between bone formation and mechanical stimulus within cortical bone: Combining 3D fluorochrome mapping and poroelastic finite element modelling
Source: Bone Rep. 2018 Feb 16;8:72–80. doi: 10.1016/j.bonr.2018.02.003 (PMC5997173; doi:10.1016/j.bonr.2018.02.003)
Supplement: Table S2 — Interstitial fluid material properties used for the 3D finite element modelling of the tibia taken from Pereira et al. (2015). [file mmc3.docx]

**Supplementary Material**

| Property |  | Units | Fluid |
| --- | --- | --- | --- |
| Specific weight | *γ* | N m^-3^ | 9.8 x 10^3^ |
| Dynamic viscosity | *μ* | Pa s | 8.9 x 10^-4^ |
| Bulk modulus | *K_f_* | MPa | 2.3 x 10^3^ |

**Table S2.** Interstitial fluid material properties used for the 3D finite element modelling of the tibia taken from Pereira et al.^(38)^
